# Supplementary material for: Novel Driver Strength Index highlights important cancer genes in TCGA PanCanAtlas patients
Source: PeerJ. 2022 Aug 11;10:e13860. doi: 10.7717/peerj.13860 (PMC9375969; doi:10.7717/peerj.13860)
Supplement: Supplemental Information 2 [file peerj-10-13860-s002.zip › COHORTS/genes plots/cohorts/2021_8_16_14_9_distribution_cohorts_males_UVM.pdf]

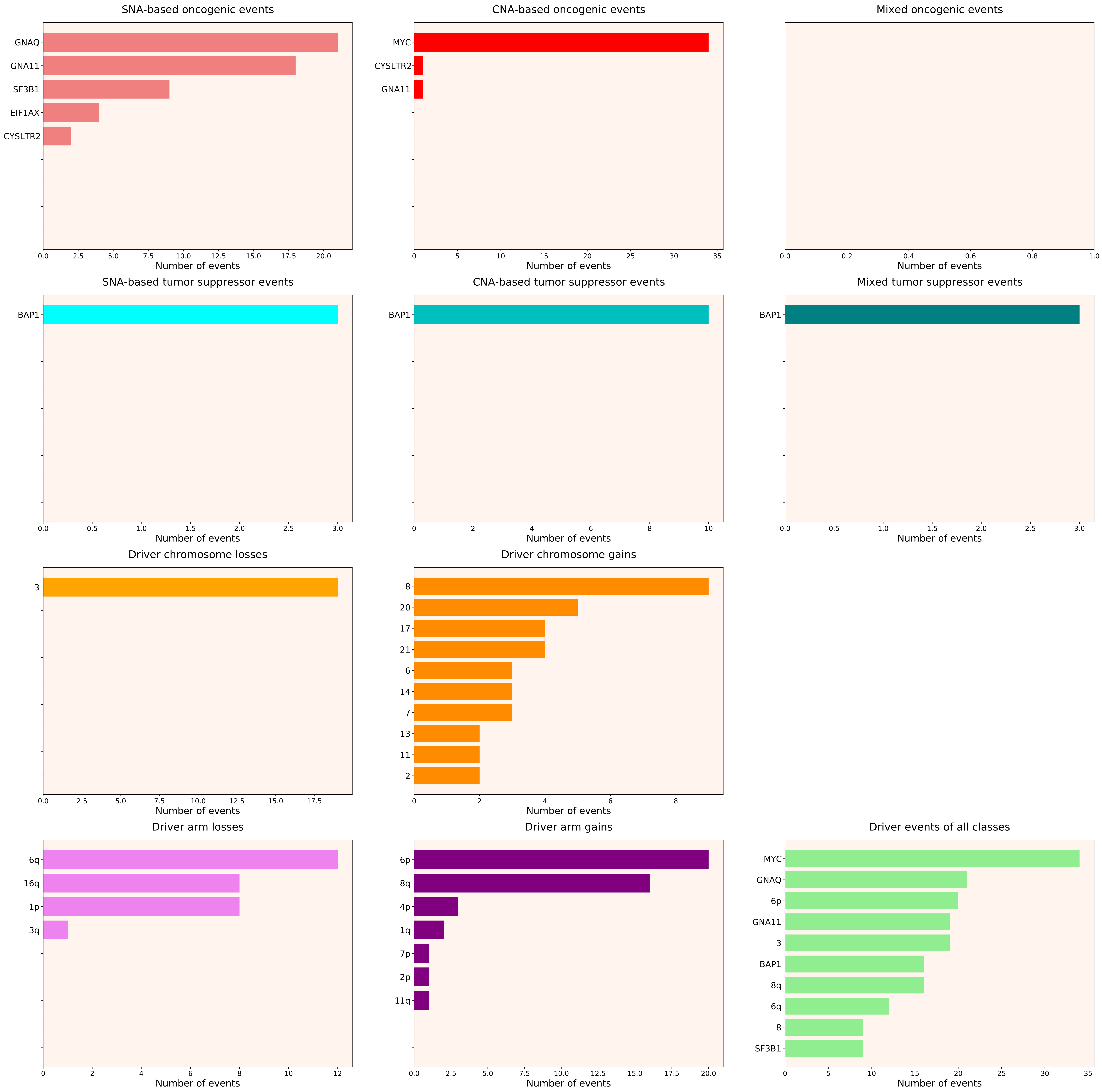

SNA-based oncogenic events
GNAQ
GNA11
SF3B1
EIF1AX
CYSLTR2
0.0 2.5 5.0 7.5 10.0 12.5 15.0 17.5 20.0
Number of events
CNA-based oncogenic events
MYC
CYSLTR2
GNA11
0 5 10 15 20 25 30 35
Number of events
Mixed oncogenic events
0.0 0.2 0.4 0.6 0.8 1.0
Number of events
SNA-based tumor suppressor events
BAP1
0.0 0.5 1.0 1.5 2.0 2.5 3.0
Number of events
CNA-based tumor suppressor events
BAP1
0 2 4 6 8 10
Number of events
Mixed tumor suppressor events
BAP1
0.0 0.5 1.0 1.5 2.0 2.5 3.0
Number of events
Driver chromosome losses
3
0.0 2.5 5.0 7.5 10.0 12.5 15.0 17.5
Number of events
Driver chromosome gains
8
20
17
21
6
14
7
13
11
2
0 2 4 6 8
Number of events
Driver arm losses
6q
16q
1p
3q
0 2 4 6 8 10 12
Number of events
Driver arm gains
6p
8q
4p
1q
7p
2p
11q
0.0 2.5 5.0 7.5 10.0 12.5 15.0 17.5 20.0
Number of events
Driver events of all classes
MYC
GNAQ
6p
GNA11
3
BAP1
8q
6q
8
SF3B1
0 5 10 15 20 25 30 35
Number of events
